# Pharmacokinetics of a Long-Acting Formulation of Oxytetracycline in Freshwater Crocodiles (*Crocodylus siamensis*) after Intramuscular Administration at Three Different Dosages

**DOI:** 10.3390/ani10081281

**Published:** 2020-07-27

**Authors:** Saranya Poapolathep, Narumol Klangkaew, Napasorn Phaochoosak, Tara Wongwaipairoj, Mario Giorgi, Narongsak Chaiyabutr, Darren J. Trott, Amnart Poapolathep

**Affiliations:** 1 Department of Pharmacology, Faculty of Veterinary Medicine, Kasetsart University, Bangkok 10900, Thailand; fvetsys@ku.ac.th (S.P.); fvetnak@ku.ac.th (N.K.); fvetanp@ku.ac.th (N.P.); 2 Wongveerakit Crocodile Farm, Bo Phloi, Kanjanaburi 71160, Thailand; tara.wongwai@gmail.com; 3 Department of Veterinary Science, University of Pisa, 56112 Pisa, Italy; mario.giorgi@unipi.it; 4 Department of Physiology, Faculty of Veterinary Science, Chulalongkorn University, Bangkok 10330, Thailand; narongsak.c@chula.ac.th; 5 Australian Centre for Antimicrobial Resistance Ecology, School of Animal and Veterinary Science, University of Adelaide, Roseworthy 5371, Australia; darren.trott@adelaide.edu.au

**Keywords:** oxytetracycline, pharmacokinetics, freshwater crocodiles, LC-MS/MS

## Abstract

**Simple Summary:**

The oxytetracycline long-acting formulation (OTC-LA) is used to treat sensitive pathogenic bacteria in the freshwater crocodile, *Crocodylus siamensis*. The pharmacokinetic profiles of differential dosages of OTC after intramuscular administration were investigated to determine the appropriate dosage for the treatment of bacterial infections in freshwater crocodiles. In freshwater crocodiles, dosages of 10 and 20 mg/kg produced OTC plasma concentrations higher than 2.0 µg/mL as a minimum inhibitory concentration (MIC) for 192 h and 216 h after intramuscular administration, respectively, while the OTC plasma concentration remained below the MIC of 2.0 µg/mL at a dosage of 5 mg/kg body weight (b.w.). When considering plasma protein binding of 32%, an intramuscular (i.m.) administration at a dosage of 10 mg/kg b.w. might be effective for two weeks to treat sensitive pathogenic bacteria in freshwater crocodiles.

**Abstract:**

To date, the necessary pharmacokinetic information has been limited to establish suitable therapeutic plans for freshwater crocodiles. Therefore, this study was conducted to evaluate the pharmacokinetic profile of the oxytetracycline long-acting formulation (OTC-LA) in the freshwater crocodile, *Crocodylus siamensis*, following a single intramuscular (i.m.) administration at three different dosages of 5, 10 and 20 mg/kg body weight (b.w.). Blood samples were collected at assigned times up to 216 h after i.m. administration at the three different dosages. The plasma concentrations of OTC were measured using a validated liquid chromatography tandem-mass spectrometry (LC-MS/MS) method. The C_max_ (± SD) values of OTC were 2.15 ± 0.51 µg/mL, 7.68 ± 1.08 µg/mL and 17.08 ± 2.09 µg/mL at doses of 5, 10 and 20 mg/kg b.w., respectively. The elimination half-life values were 33.59 ± 2.51 h, 38.42 ± 5.47 h and 38.04 ± 1.98 h at dosages of 5, 10 and 20 mg/kg b.w., respectively. Based on the pharmacokinetic data, the pharmacokinetic/pharmacodynamic (PK/PD) index, the susceptibility break-point and plasma protein binding, a dosage once every two weeks of 10 mg/kg b.w. OTC intramuscularly might be suitable for initiating the treatment of susceptible bacterial infections in freshwater crocodiles. However, further PK/PD studies are warranted to confirm whether the dose rates used in this study can produce longer-term antimicrobial success for diseases caused by susceptible bacteria in freshwater crocodiles.

## 1. Introduction

Oxytetracycline (OTC), a tetracycline derivative obtained from *Streptomyces rimosus*, is a broad-spectrum antibiotic used against a variety of pathogens, including bacteria, mycoplasma, rickettsia, chlamydiae and even some protozoa [1]. OTC is a bacteriostatic antibiotic that inhibits protein synthesis by reversibly binding to the 30S ribosomal subunit of a susceptible organism [2]. The antibacterial efficacy of OTC is described as time-dependent. OTC is distributed rapidly and extensively in animal body fluids and tissues, undergoes enterohepatic recirculation in some mammals and is then excreted primarily by glomerular filtration and biliary elimination [3].

One of the major obstacles to the successful treatment of infectious diseases in freshwater crocodile species is the incorrect dosing of antibiotics. When drugs are administered at subtherapeutic levels, there is an increased risk of therapeutic failure or the development of bacterial resistance or both. The treatment of bacterial infections should be based on a rational scientific approach, which, in the case of antibiotics, is the pharmacokinetic/pharmacodynamic (PK/PD) approach. In breeding farms, crocodile mortality is often directly related to infectious diseases precipitated by stress [4]. Currently, there are few reports on the pharmacokinetics and dosage regimen of antimicrobial drugs in crocodiles. Regarding pharmacokinetic profiles, a number of antimicrobial drugs have been investigated in American alligators, estuarine crocodiles and freshwater crocodiles [5,6,7,8,9,10,11].

Long-acting formulations of antimicrobial agents are advantageous for the clinical treatment of dangerous animal species. Thus, it is valuable to examine whether therapeutic concentrations of OTC can be maintained for a long period in freshwater crocodiles. The term “long-acting” implies that the formulation provides a prolonged circulation of antibacterial concentrations of the active agent and should show a commensurate improvement in clinical efficacy [12]. In American alligators, therapeutic plasma concentrations of OTC were maintained for seven days after intramuscular (i.m.) administration at a dosage of 10 mg/kg body weight (b.w.) [5]. Therefore, the aim of this study was to evaluate the pharmacokinetic profiles of a long-acting formulation of oxytetracycline (OTC-LA) in the freshwater crocodile, *Crocodylus siamensis*, following an intramuscular (i.m.) administration at three different dosages of 5, 10 and 20 mg/kg b.w.

## 2. Materials and Methods

### 2.1. Animals

Fifteen freshwater crocodiles, *C. siamensis* (females, aged 2.5–3.2 years, body weights of 14.8–20.4 kg and lengths of 140–166 cm), were used in the study. All 15 experimental farmed crocodiles were housed in cement ponds at Wongveerakit Farm, Bo Phloi Kanchanaburi Province, Thailand. The cement pond area is 5 m (wide) and 10 m (length), including slope and shelter. Five experimental crocodiles were house per each pond. The water was changed every day through the experimental period to clear the excreted drug and waste. All experimental procedures were performed according to the Guidelines for Animal Experiments and approved by the Animal Ethics Research Committee of the Faculty of Veterinary Medicine, Kasetsart University, Bangkok, Thailand. The experiment was conducted within an environmental temperature range of 27–33 °C. Prior to inclusion in the study, each animal was deemed healthy based on their health history, physical examination and complete blood count analysis, all of which were performed within 2 days of the commencement of the study. Body weights for the purpose of the dose calculation were acquired no more than 48 h prior to drug administration.

### 2.2. Drugs and Chemicals

Oxytetracycline-long acting formulation (Terramycin/LA^®^) for injection was purchased from Zoetis (Zoetis Inc., New Jersey, NJ. USA). OTC of analytical standard was purchased from Sigma Chemical Co. (St. Louis, MO, USA). Other reagents and chemicals of analytical grade were purchased from Sigma Chemical Co. (St. Louis, MO, USA). Purified water was produced using the Milli-Q water purification system from Millipore, Inc. (Bedford, MA, USA). OTC was dissolved with 0.01% formic acid in 50% acetonitrile solution for spiked samples and calibration.

### 2.3. Experimental Design

The 15 crocodiles were weighed and identified using a permanent numbered marker on the back region of the head and divided into three groups (*n* = 5) using a randomization procedure according to a parallel study design. Groups 1, 2 and 3 were administered OTC-LA i.m. at dosages of 5, 10 and 20 mg/kg b.w., respectively. The dose rates used in this study were based on preliminary studies in the target species and a previous study in American alligators [5]. Animals were manually restrained for the blood collection period between dosing, and two hours later, animals were left tied with their eyes covered to calm them. For the later blood collections, animals were released and recaptured. The i.m. dosing was delivered into the left biceps using a 22 gauge, 1.5-inch-long needle. The injection site was disinfected using ethyl alcohol prior to injection. The i.m. injection site was selected to avoid the renal portal system first-pass effect. Blood samples (1.5–2.0 mL) were collected from the tail vein of each animal in heparinized tubes at 0, 5, 15 and 30 min and at 1, 2, 4, 6, 8, 12, 24, 48, 72, 96, 120, 144, 168, 192 and 216 h after drug administration. The plasma was separated using centrifugation (1986× *g*) for 15 min, harvested and immediately stored at −20 °C for 2 weeks before analysis.

### 2.4. Sample Extraction Procedure

The OTC extraction method was performed as previously described [13,14] and revalidated in spiked plasma of the crocodiles. Briefly, 500 µL of freshwater crocodile plasma was deproteinated by adding 50 µL of 15% trichloroacetic acid in acetonitrile (ACN) in a 1-mL conic Eppendorf tube and then vortexed for 1 min. The mixture was centrifuged at 7168× *g* for 15 min at 4 °C. The supernatant was collected into new plastic vials. The supernatant was collected, passed through a 0.22-μm nylon syringe filter and subjected to liquid chromatography-tandem mass spectrometry (LC-MS/MS).

### 2.5. LC Parameters

The LC analysis was performed using an Agilent 1200 series system consisting of a binary high-pressure-gradient pump, a vacuum solvent degassing unit, an automatic sample injector and a column thermostat (Agilent Technologies, Waldbronn, Germany). Separation was achieved using a ZORBAX Eclipse Plus Rapid Resolution HT (RRHT) C18 column (4.6 × 50 mm, 1.8-μm particle size, Agilent Technologies) with a guard column (4.6 × 5 mm, 1.8-μm particle size, Agilent Technologies). The column was maintained at a temperature of 35 °C. The LC mobile phase program consisted of a binary gradient of 0.1% formic acid in Milli-Q water (mobile phase A) and acetonitrile (mobile phase B). The gradient conditions were: 0–6.0 min, from 95% to 35% A, 6.0–7.0 min, 5% A, 7.0–10.0 min, 5% A and 10.0–11.0 min, from 5% to 95% A, followed by re-equilibration at 95% A until 15 min. The flow rate was 400 µL/min, and the injection volume was 5 µL.

### 2.6. MS Parameters

Mass spectrometry was performed using an Agilent Technologies 6460 triple quadrupole mass spectrometer equipped with an electrospray ionization (ESI) source and Agilent Mass Hunter Workstation Software version 1.2 (Agilent Technologies, Waldbronn, Germany). ESI-MS/MS was operated at unit mass resolution in the multiple reaction monitoring positive ion mode with the following settings: nebulizer gas pressure 45 psi, gas flow 5.0 L/min, gas temperature 300 °C, sheath gas temperature 380 °C, sheath gas flow 10 L/min and capillary voltage 3500 V. The following transitions were used: OTC: *m/z* 461.0 > 443.0 and 461.0 > 426.0.

### 2.7. Validation Procedure

The calibration standard concentrations were prepared by spiking the working standard solution into crocodile blank plasma to yield final concentrations of 0.05, 0.1, 0.5, 1, 5, 10 and 20 µg/mL. The coefficient of determination (*r*^2^) value of the OTC calibration curves was 0.999. Seven duplicates of the quality control (QC) sample at concentrations of 0.1, 1, 10 and 20 µg/mL were prepared and used to determine the recoveries, intra-day and inter-day precision and accuracy of the method. The procedure was repeated five times within the same day to gain intra-day run precision and accuracy and five times for each concentration over five different days to obtain inter-day run precision and accuracy. The extraction recoveries were 86.82% ± 3.42%, 89.66% ± 4.12%, 92.82% ± 2.45% and 95.42% ± 2.12% for 0.1, 1, 10 and 20 µg/mL, respectively. The inter-day precision and accuracy ranged from 3.42% to 6.88% and from 92.65 to 98.26%, respectively. Levels for the limit of detection (LOD) and the limit of quantification (LOQ) of OTC were 0.01 and 0.05 µg/mL, respectively.

### 2.8. Plasma Protein Binding Assay

Protein biding was determined using ultracentrifugation (Optima^TM^ Max-XP, Beckman Coulter, Inc.,Indianapolis, IN, USA) [15]. Fresh OTC-free plasma samples from the freshwater crocodiles and phosphate-buffered saline were added to known concentrations of OTC, ranging from 1 to 10 µg/mL. Samples were centrifuged at 480,000× *g* for 2.5 h. Each plasma sample and its corresponding ultrafiltrate were assayed using LC-MS/MS, as described above. The percentage of the plasma protein-binding fraction was calculated according to the following equation:

Protein binding (%) = total concentration—ultrafiltrate concentration/total concentration (×100)

### 2.9. Pharmacokinetic Analysis

The concentration of OTC in experimental crocodiles with respect to time was analyzed using a noncompartmental model (ThothPro™ 4.3.0 v software, ThothPro LLC, Poland). The elimination half-life (t_1/2__λz_) was calculated using a nonlinear least squares regression analysis of the concentration-time curve, and the areas under the curve (AUC) were calculated using the linear-up log-down rule to the final concentration time point (Ct). From these values, the mean residence time (MRT = AUMC/AUC), C_max_/dose and AUC/dose were determined. Individual values between AUC_0-__∞_ and AUC_0-t_ were lower than 20% of AUC_0-__∞_, with *r*^2^ > 0.85 for the terminal phase regression line (at least 3 points were used for this latter estimation).

### 2.10. Statistical Analysis

Pharmacokinetic variables were evaluated using one-way ANOVA analysis and the Tukey’s test for multiple comparisons to determine statistically significant differences among groups. Both pharmacokinetic parameters and OTC plasma concentrations were presented as mean ± SD (normality tested using the Shapiro-Wilk test). All analyses were conducted using GraphPad Prism version 5.0 (GraphPad Software, La Jolla, CA, USA). Differences were considered significant for *p* < 0.05.

## 3. Results

No adverse effects at the point of injection and no behavioral or health alterations were observed in the experimental animals during or after i.m. administration of OTC at the three different dosages.

**Figure 1:**
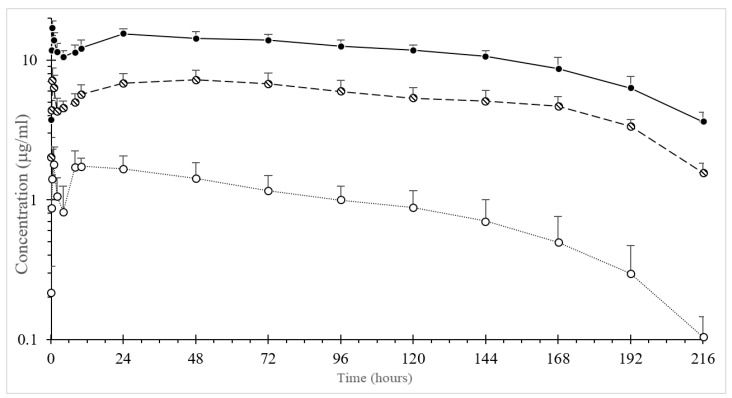
Semi-log average (± SD) plasma concentration versus time curve of long-acting formulation oxytetracycline after intramuscular administration at three different dosages of 5 mg/kg body weight (b.w.) (-○-), 10 mg/kg b.w. (-ϴ-) and 20 mg/kg b.w. (-●-) in the freshwater crocodile, *Crocodylus siamensis* (*n* = 5). Error bars indicate standard deviation.

OTC was quantifiable up to 216 h in all treated groups. The plots of the mean plasma concentration-time curves of OTC are displayed in Figure 1. The pharmacokinetic profiles had a double peak in all the animals, irrespective of treatment. The first peak appeared at 1.0, 0.5 and 0.5 h after i.m. administration at dosages of 5, 10 and 20 mg/kg b.w., respectively. After rapid absorption followed by a significant decline in the concentration, a second peak was observed at 48 h after dosages of 5 and 10 mg/kg b.w. and at 24 h after a dosage of 20 mg/kg b.w. The plasma of OTC then gradually declined.

The value for t_1/2__λz_ was significantly shorter in the 5 mg/kg b.w. group compared to the 10 and 20 mg/kg b.w. groups, where the results were almost identical. The mean (± SD) pharmacokinetic parameters of OTC are reported in Table 1. The average value of the plasma protein-binding percentage of OTC was 32.69% ± 9.16%.

A linear relationship between dose and AUC or C_max_ was found between 10 and 20 mg/kg b.w. but not for the 5 mg/kg b.w. treatment.

## 4. Discussion

The study was designed to characterize the pharmacokinetic features of OTC-LA after i.m. administration at dosages of 5, 10 and 20 mg/kg b.w. in the freshwater crocodile, *C. siamensis*. A few published papers have described the pharmacokinetics of antibacterial drugs in reptilian species—namely, American alligators, estuarine crocodiles and freshwater crocodiles [5,6,7,8,9,10,11]. However, to date, there have been no reports on the pharmacokinetics of OTC in the freshwater crocodile, *C. siamensis*. In addition, drug dosages in freshwater crocodiles are often extrapolated from domestic animals or members of similar taxonomic orders, but even careful allometric scaling based on similar species can result in unpredictable pharmacokinetic profiles and toxicoses [16]. In the present study, the critical pharmacokinetic parameters were evaluated after i.m. administration at three different dosages.

Plasma concentrations after OTC-LA administration were quantifiable up to 216 h after i.m. administration at dosages of 5, 10 and 20 mg/kg b.w. OTC was rapidly detected in plasma, with a first peak at 0.5–1.0 h; then, the concentration dropped rapidly, and subsequently, a second peak (24–48 h) occurred, followed by the gradual decline of the drug in the plasma circulation. This profile has been earlier reported in alligators [5] and fish [17]. In fish, it was speculated to have been due to drug reabsorption via the skin, but this was unlikely in the present study, because crocodile skin is very hard for a drug to penetrate [17], and any drug eliminated in the water would have been diluted at a negligible concentration due to the large volume of water in the holding pond. In alligators [5], this peculiar PK profile has been hypothesized to be due to OTC-LA formulation, with a portion acting immediately (the first peak), followed by a depot effect (the second peak) [5]. The ability of OTC-LA to generate inflammation in the tissue is injected. In such a case, the OTC might be rapidly adsorbed until the inflammation takes place; then, due to homeostasis, the drug is no longer able to enter the blood circulation at the same rate; then, as the inflammation is slowly resolved, the drug concentration rises again (the second peak).

The t_1/2λ_ value of OTC obtained in the freshwater crocodiles was long and almost identical following i.m. administration after two dosages of 10 and 20 mg/kg b.w. This indicated that the overall rate of elimination of OTC in freshwater crocodiles was slow. The long t_1/2λz_ reported here was consistent with the low elimination rate constant and the slow clearance rate. However, the mean t_1/2λz_ value was shorter than that reported in American alligators (approximately 131 h) [5]. These differences might have resulted from species-specific differences, different environmental temperatures during the experiment, the time of blood collection or different sensitivities in the analytical methods [18]. Accordingly, the MRT values in the present investigation were shorter than those reported in American alligators (254 h) [5]. In the present study, the average percentage of protein binding of OTC in the freshwater crocodile plasma was moderate (32.69%), although, to date, no comparative value for this parameter has been reported in reptiles. The pharmacokinetics of OTC in this species are dose-dependent, between 10 and 20 mg/kg. In contrast, a nonlinear relationship between the dose and AUC or C_max_ was shown with the 5 mg/kg dose. Further studies are warranted to explain this finding.

High plasma OTC concentrations were found in freshwater crocodiles after i.m. administration at dosages of 10 and 20 mg/kg b.w. The MIC value for OTC is about 0.25–2.0 µg/mL for most susceptible microorganisms that can cause significant diseases in animal species [19]. The Clinical Laboratory Standard Institute (CLSI) suggests a cut-off value ranging from 0.25 to 2.0 µg/mL for tetracycline susceptibility depending upon both the bacterial (mammalian pathogens) and animal species (mammalian). CLSI standards for the interpretation of bacteria isolated from animals does not contain breakpoints for the interpretation of susceptibility test results for OTC in reptiles. Due to inter- and intraspecies differences between animals, it is necessary to specify the dosage regimen for the prudent use of antibiotics in each animal species. The AUC/MIC ratio can be used as a predictor of antibacterial success in clinical subjects, since OTC is classified as a bacteriostatic agent [20]. Studies performed in other animal species show that a favorable therapeutic target is AUC/MIC > 25. Bacteria considered “susceptible” in other animals have MIC values of 2 µg/mL or less [19]. Based on this value, the dose administered to crocodiles reached the target for AUC (truncated to 24 h) for bacteria with MIC values ≤ 3.8 and 8.6 µg/mL for 10 and 20 mg/kg b.w., respectively. Although the AUC/MIC ratio achieved in this study was high (ratio > 900 for the highest dose), this was the entire AUC to infinity. AUC/MIC targets for mammals are calculated for only a 24-h interval, whereas the concentrations in this study were above a MIC value of 2 µg/mL for over 15 days. All the PK/PD calculations were performed considering the percentage protein binding of the OTC obtained in this study. Studies on clinically infected crocodiles warranted a confirmation of this data. Notably, the OTC plasma concentration was not achieved and did not remain above the MIC of 2.0 µg/mL at a dosage of 5 mg/kg b.w. However, the MIC values of the important bacterial pathogens that affect freshwater crocodiles should be examined for the efficacious use of OTC in freshwater crocodiles.

## Figures and Tables

**Table 1:** Mean ± SD values of the pharmacokinetic parameters of the long-acting formulation of oxytetracycline (OTC-LA) following intramuscular administration at three different dosages (5, 10 and 20 mg/kg body weight (b.w.)) in the freshwater crocodile, *Crocodylus siamensis* (*n* = 5 per group).

Pharmacokinetic Parameter (Unit)	OTC-LA		
	5 mg/kg	10 mg/kg	20 mg/kg
C_max_ (µg/mL)	2.15 ± 0.51	7.68 ± 1.08 ^ac^	17.08 ± 2.09 ^ab^
T_max_ (h) ^§^	1.0 (0.5–8.0)	0.5 (0.5–1.0)	0.50 (0.00)
λ_z_ (h^−1^)	0.021 ± 0.001	0.018 ± 0.002	0.018 ± 0.001
t_1/2λ_ (h)	33.59 ± 2.51	38.42 ± 5.47 ^a^	38.04 ± 1.98 ^a^
AUC_last_ (h mg/L)	199.27 ± 51.91	1101.36 ± 164.54 ^ac^	2405.15 ± 182.37 ^ab^
AUC_0-∞_ (h mg/L)	206.96 ± 54.56	1196.05 ± 159.93 ^ac^	2611.76 ± 196.08 ^ab^
C_max_/dose	0.43 ± 0.10	0.77 ± 0.11	0.85 ± 0.10
AUC/dose	39.85 ± 10.38	110.14 ± 16.45 ^a^	120.26 ± 9.12 ^a^
MRT_last_ (h)	76.36 ± 8.79	93.55 ± 2.72 ^a^	92.03 ± 3.01 ^a^

Note: C_max_ = peak plasma concentration, T_max_ = time of peak concentration, λz = terminal phase rate constant, t_1/2λ_ = terminal half-life, AUC_last_ = area under the curve from 0 to the last point of drug quantification, AUC_0-∞_ = area under the curve from 0 h to infinity and MRT_last_ = mean residence time from 0 to the last point of drug quantification. *p* < 0.05; ^a^ = significant difference from the group that received OTC-LA intramuscularly at a dosage of 5 mg/kg b.w., ^b^ = significant difference from the group that received OTC-LA intramuscularly at a dosage of 10 mg/kg b.w. and ^c^ = significant different from the group that received OTC-LA intramuscularly at a dosage of 20 mg/kg b.w. ^§^ = median value and range.
